# ADDICTION AND THE EVOLUTION AND COMPLIANCE OF SCHIZOPHRENIC PATIENTS

**DOI:** 10.1192/j.eurpsy.2023.2237

**Published:** 2023-07-19

**Authors:** F. Azraf, H. Chebli, F. El Omari

**Affiliations:** Arrazi Hospital, Faculty of Medicine and Pharmacy of Rabat, Sale, Morocco

## Abstract

**Introduction:**

Schizophrenia is a chronic illness, affecting approximately 1% of the general population. The more chronic a treatment is, the poorer the quality of adherence. In fact, approximately 60% of patients with schizophrenia are incompletely or non-adherent within one year of the first episode. Poor adherence has consequences in terms of clinical status, quality of life and psychosocial functioning due to associated relapses. One of the main factors of poor compliance is the use of addictive substances. Half of schizophrenic patients have, or have had, an addictive comorbidity during their life. Moreover, schizophrenic patients with addictive comorbidity have a more severe symptomatology and have more associated physical, psychological and social problems, representing a challenge in the management

**Objectives:**

The objective of our work is to evaluate the impact of addictive comorbidity on medication adherence and relapse.

**Methods:**

This is a cross-sectional study, carried out in patients with schizophrenia hospitalized or followed in ambulatory consultation, at the psychiatric hospital Ar-razi of Salé. The data collection (sociodemographic, clinical and therapeutic) is carried out by a questionnaire established for this purpose. The use of substances is evaluated according to the DSM-V criteria

**Results:**

A total of 110 schizophrenic patients were followed or hospitalized, of which 61.8% had a substance use disorder. The average age of onset of substance use was 18 years. The duration of substance use ranged from 1 to 11 years and up to 49 years for tobacco and cannabis. 72% of the addicts tried to stop their use alone or in outpatient settings, only 17% in inpatient settings. The average duration of abstinence was 11 months and ranged from 1 to 18 years. 81% reported remission of the disease after abstinence. The hospitalization rate was 88.2% for addicts versus 85% for non-addicts. The number of hospitalizations was 180 for addicts versus 78 for non-addicts. The cumulative duration of hospitalization is 208 months against 96 months for addicts. The duration of remission is 5 months for addicts and 24 months for non-addicts. 71% of non-addicts patients have well observed their treatment against 57% of addicts

**Conclusions:**

The weight of co-morbid addictions represents between 1/5 and 1/3 of the factors at stake in the compliance and the risk of relapse of patients suffering from schizophrenia. The development of specific care strategies for a global management of addiction and schizophrenia should be a priority.

**Disclosure of Interest:**

None Declared

